# Annotations

**Published:** 1894-09-29

**Authors:** 


### Annotations.

### The Diphtheria Problem.

Seeing that it is proved beyond dispute that there has been an enormous increase of diphtheria in large towns during the last twenty years, and that it is equally obvious there ought to have been a diminution if the science of medicine be so progressive as we are constantly affirming and hoping that it is, we ought, as the natural custodians of the public health, to put ourselves upon new inquiries and fresh action. The massing together of children in elementary schools may be, and probably is, accountable for a portion of the increase. But are there no other contributing factors? It has been suggested that the free opening and ventilation of sewer drainage into the streets of towns, which has been so extensively developed during the past two or three decades, may be one of the causes of the enormous multiplication of the diphtheria bacillus in the air, and on exposed surfaces in streets and houses. This seems highly probable, and, if it should prove to be the case, there would appear to be no difficulty in reducing such a danger to a minimum, except, of course, the difficulty of cost. The remedy for such a state of things is the destruction of the odorous substances which are given off by the drains into the atmosphere. This may easily be accomplished by fire or other methods. Some experiments in destruction have, we believe, been quite recently made at Tottenham, and the successful destruction of vile odours, with the resultant sweetening of the atmosphere, has been quite remarkable. As yet no facts have been published, but the experiments have demonstrated that sewer gas can very easily be destroyed, and it is highly probable that with its extensive destruction one cause of the alarming increase of diphtheria in our large towns would be removed.

### The Effects of Creasote upon Tuberculosis.

A great many medical men now prescribe creasote in tuberculosis, and many of them will no doubt be glad to have some experimental facts which may guide them in its rational use. Does creasote affect the tubercle bacillus, or does it affect the patient, or does it affect both? According to some recent experiments made by Dr. Kington Fyffe it affects both the patient and the tubercle bacilli; moreover, it influences both favourably, and, when properly employed, very favourably. There are two well-known methods of administering creasote to tuberculous patients, and one or two less well known. The two methods commonly employed are the giving of the drug by the mouth and its exhibition as an inhalant. Theorists would have us believe that, inasmuch as tuberculosis is most frequently located in the lungs, inhalation must be nature's rational method of dealing with phthisis in the lung. But Dr. Fyffe's experiments point to a totally different conclusion. Creasote given by the mouth is much more effective than creasote inhaled from a bottle or sponge. It influences both the patient and the local lesion much more powerfully, and in strict proportion to the dose administered. A third method employed was Dr. Arnold Chaplin's method of saturating a chamber full of hot air with creasote and making the patient sit in it. This proved remarkably effective. But the most striking results of all followed upon the injection of creasote subcutaneously. The experiments were made on guinea pigs artificially infected with tubercle. Against the subcutaneous method is the objection that some of the guinea pigs died of cellulitis set up by the injected creasote. The practical conclusions are, that creasote is of great service in early phthisis; that it is of very much greater service when taken by the mouth than when inhaled by the methods in common use; that it is necessary to give it in as full doses as can be borne; and that when all is done it only prolongs life, but does not finally cure the disease.

### The Lighthouse Keepers' Grievance.

Since we called attention on the 24th ult. to the hardships endured by lighthouse men with the sanction of the Trinity House and the Board of Trade, much attention has been directed to the matter in the Press. The *Daily Telegraph*, the *Daily News*, *Daily Graphic*, *Evening Standard*, the *Westminster* and *St. James's Gazettes*, the *Globe*, and the *Sun*, in addition to many provincial papers, have published much information, and proved the case against the Trinity Brethren and the Board of Trade up to the hilt. The *Manx Sun* of the 15th inst. publishes the various petitions which the lighthouse keepers have addressed to their employers, which prove that reforms are called for throughout the lighthouse service. On August 15th, 1892, the Trinity House informed the men that the Board of Trade were considering whether they could give effect to the prayer of the petitioners, but that owing to the change of Ministry, a decision could not be arrived at until the autumn. It is fair to conclude therefore that the complaints respecting the men's wages, length of hours, short leave, promotion and holidays, the visits of friends and age of retirement are well founded, and that they demand and should receive prompt attention. The autumn of 1892 is long past and gone, but no reply from the Board of Trade is yet forthcoming. To the surprise of the public, instead of meeting the statements made in the Press on behalf of the men frankly and fully the Elder Brethren of the Trinity House have striven to evade the plain issues at stake to their own discredit. The grievances of the men are so genuine and their requests so moderate that the Trinity Brethren and the Board of Trade must either deal with the case promptly and generously, or Parliament will certainly bring pressure to bear upon both. There is evidence in proof of this contention, as one correspondent points out, seeing that the Irish Members kept the subject before the Board of Trade until the Irish lighthouse keepers' grievances were redressed. In many cases the proved abuses connected with the English and Scotch lighthouse services cannot longer be ignored by those responsible, and we shall hope to hear that both the Elder Brethren and the Board of Trade have spontaneously remedied them as they ought to have done long ago.

